# The Regulatory Repertoire of *Pseudomonas aeruginosa* AmpC ß-Lactamase Regulator AmpR Includes Virulence Genes

**DOI:** 10.1371/journal.pone.0034067

**Published:** 2012-03-29

**Authors:** Deepak Balasubramanian, Lisa Schneper, Massimo Merighi, Roger Smith, Giri Narasimhan, Stephen Lory, Kalai Mathee

**Affiliations:** 1 Department of Biological Sciences, College of Arts and Science, Florida International University, Miami, Florida, United States of America; 2 Molecular Microbiology and Infectious Diseases (Herbert Werthiem College of Medicine), Florida International University, Miami, Florida, United States of America; 3 Microbiology and Molecular Genetics, Harvard Medical School, Boston, Massachussetts, United States of America; 4 School of Computing and Information Science, College of Engineering and Computing, Florida International University, Miami, Florida, United States of America; Vrije Universiteit Brussel, Belgium

## Abstract

In *Enterobacteriaceae*, the transcriptional regulator AmpR, a member of the LysR family, regulates the expression of a chromosomal β-lactamase AmpC. The regulatory repertoire of AmpR is broader in *Pseudomonas aeruginosa*, an opportunistic pathogen responsible for numerous acute and chronic infections including cystic fibrosis. In addition to regulating *ampC*, *P. aeruginosa* AmpR regulates the sigma factor AlgT/U and production of some quorum sensing (QS)-regulated virulence factors. In order to better understand the *ampR* regulon, we compared the transcriptional profile generated using DNA microarrays of the prototypic *P. aeruginosa* PAO1 strain with its isogenic *ampR* deletion mutant, PAOΔ*ampR*. Transcriptome analysis demonstrates that the AmpR regulon is much more extensive than previously thought, with the deletion of *ampR* influencing the differential expression of over 500 genes. In addition to regulating resistance to β-lactam antibiotics via AmpC, AmpR also regulates non-β-lactam antibiotic resistance by modulating the MexEF-OprN efflux pump. Other virulence mechanisms including biofilm formation and QS-regulated acute virulence factors are AmpR-regulated. Real-time PCR and phenotypic assays confirmed the microarray data. Further, using a *Caenorhabditis elegans* model, we demonstrate that a functional AmpR is required for *P. aeruginosa* pathogenicity. AmpR, a member of the core genome, also regulates genes in the regions of genome plasticity that are acquired by horizontal gene transfer. Further, we show differential regulation of other transcriptional regulators and sigma factors by AmpR, accounting for the extensive AmpR regulon. The data demonstrates that AmpR functions as a global regulator in *P. aeruginosa* and is a positive regulator of acute virulence while negatively regulating biofilm formation, a chronic infection phenotype. Unraveling this complex regulatory circuit will provide a better understanding of the bacterial response to antibiotics and how the organism coordinately regulates a myriad of virulence factors in response to antibiotic exposure.

## Introduction

*Pseudomonas aeruginosa* is one of the leading opportunistic Gram-negative nosocomial pathogens. This is particularly true in critically ill patients, where multi-drug resistant *P. aeruginosa* is a severe problem. It is the leading pathogen in ventilator-associated pneumonia with a mortality rate of 40–60% [1]. *P. aeruginosa* is also a primary cause of urinary tract infections in the US and Europe [2], wound infections leading to bacteremia with one-third to two-thirds mortality rate [3], [4], pulmonary infections including cystic fibrosis (CF) [5], lung cancer patients [6] and in pediatric and adult AIDS patients [7]. Inability to eradicate the infection is partly due to intrinsic and acquired antibiotic resistance of *P. aeruginosa*. Antibiotic resistant isolates of *P. aeruginosa* are selectively favored *in vivo* in CF patients [8], [9]. Resistance of *P. aeruginosa* to the β-lactam class of antibiotics, currently used to treat *P. aeruginosa* infections, is partly mediated by a group of genes belonging to the *amp* system.

The *amp* genes were first discovered in *Enterobacter cloacae* to confer resistance to β-lactams [10] and later in other members of *Enterobacteriaceae* [11], [12], [13], [14]. The products of *amp* genes in *E. cloacae* and other organisms include the AmpC β-lactamase, the AmpG permease, a putative AmpE permease, the AmpD cytoplasmic amidase, and the transcriptional regulator AmpR [10], [11], [12], [13], [14]. Recent studies have identified another permease, AmpP that is required for β-lactamase induction in *P. aeruginosa* [15]. Expression of *ampC* is regulated by AmpR. The *ampR* gene is located adjacent to *ampC* and is divergently transcribed in *C. freundii* and *E. cloacae*, as well as in *P. aeruginosa* [16], [17], [18]. AmpR of *C. freundii* and *E. cloacae* can cross-complement each other [19] and *P. aeruginosa* AmpR is similar to that found in *C. freundii* (58%) and *E. cloacae* (62%) [20]. In *C. freundii*, AmpR binds to a 15 bp sequence 5′ TCTGCTGCAAATTT 3′ [16], [20] and there is a nearly identical putative AmpR binding site (5′ TCTGCTCCAAATTT 3′) in the *ampR*-*ampC* intergenic region in *P. aeruginosa* [21]. AmpR has a helix-turn-helix motif that is typical of DNA-binding proteins and the *C. freundii* AmpR binds DNA using this motif [16]. The AmpR-AmpC system is also conserved in many other pathogens including *Burkholderia cenocepacia* [22], *Yersinia enterocolitica* [23], and *Stenotrophomonas maltophilia* [24].

AmpR belongs to the LysR family of transcriptional regulators that typically autorepress their own expression [25] which has been demonstrated in *C. freundii* [16]. In *P. aeruginosa*, however, there is no evidence of autoregulation [21]. It has been postulated that the signals mediating *ampC* regulation by AmpR are peptidoglycan degradation products that function as effector molecules and are brought into the cell cytoplasm from their point of origin in the periplasm via the AmpG permease [26]. *In vitro* studies have demonstrated that *C. freundii* AmpR can both activate and repress *ampC* expression depending on its interaction with specific peptidoglycan degradation products [27]. Thus the levels of these cell wall intermediates dictate AmpR regulation of *ampC* and the known regulatory repertoire of AmpR in Enterobacteriaceae have been limited to regulating *ampC* expression [11], [26], [27]. Previous studies comparing the properties of *P. aeruginosa* PAO1 with its isogenic *ampR* insertion mutant, PAO*ampR*::*aacC1*, have shown that AmpR regulates *ampC* as well as some quorum sensing (QS) genes [21]. This led us to hypothesize that the regulatory role of *P. aeruginosa* AmpR is more extensive than previously thought.

To test the hypothesis that AmpR regulates different pathways in *P. aeruginosa* and to identify the AmpR regulon, we compared the expression profile of wild-type PAO1 and that of an in-frame *ampR* deletion mutant, PAOΔ*ampR*, with and without sub-MIC β-lactam stress. Our data suggests that *P. aeruginosa* AmpR is a master regulator affecting the expression of over 500 genes. Functional analyses demonstrate the negative regulatory role of AmpR of multiple virulence mechanisms including biofilm formation and the MexEF-OprN multidrug efflux pump. Further, we demonstrate that AmpR positively regulates multiple acute virulence factors. Using a *C. elegans* model, we demonstrate that AmpR is required for pathogenesis in *P. aeruginosa*. This study establishes the critical regulatory role that AmpR plays in antibiotic resistance, virulence and general metabolism in *P. aeruginosa*.

## Results

### A. Deletion of ampR reduces β-lactam resistance of PAO1

**Figure 1:**
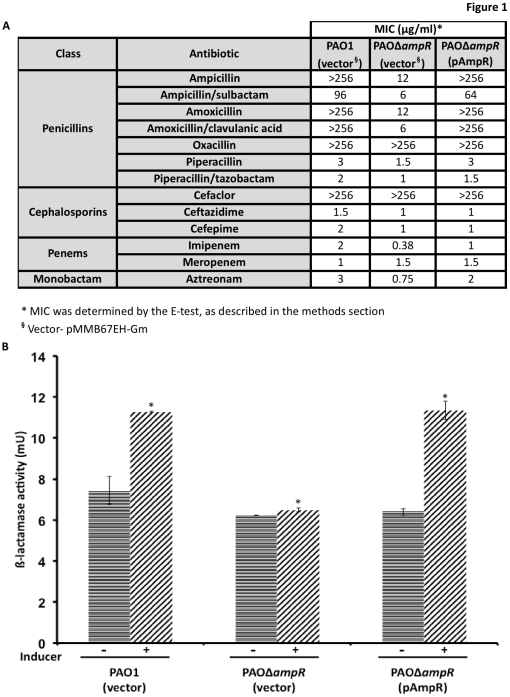
Antibiotic resistance profile of PAOΔ*ampR*. A clean in-frame deletion of *ampR* was generated in *P. aeruginosa* PAO1 as described in the methods section to generate PAOΔ*ampR*. Figure 1A shows the resistance profile of the strains to the four major classes of β-lactam antibiotics. Representative data from three different biological replicate trials are shown. The amount of β-lactamase produced was quantified (Fig. 1B) in the presence (+) and absence (−) of sub-MIC concentration of a β-lactam inducer.

In contrast to previous studies that looked at the role of *P. aeruginosa* AmpR using an insertion mutant, this study employed PAOΔ*ampR*, an in-frame deletion mutant in the prototypic *P. aeruginosa* PAO1. The presence of the *ampR* deletion was confirmed by PCR and restriction digestion of the amplicons (data not shown). AmpR is a known positive regulator of the chromosomal AmpC β-lactamase in different bacterial species [12], [20],[28]. Consequently, after constructing PAOΔ*ampR*, the strains were tested for altered production of β-lactamase. The resistance profile of β-lactam antibiotics for PAO1, PAOΔ*ampR* and PAOΔ*ampR* (pAmpR) shows that, as expected, loss of *ampR* enhances strain sensitivity to β-lactams and expressing *ampR* in *trans* on a low-copy plasmid can restore this defect with multiple β-lactam antibiotics (Fig. 1A). Loss of *ampR* seems to have a stronger effect on penicillins (ampicillin, amoxicillin and piperacillin, with and without β-lactamase inhibitors), imipenem and tazobactam than the cephalosporins tested. This finding is interesting because AmpC is a cephalosporinase. Overexpression of *ampC* under P*_tac_* control, however, results in enhanced resistance to the cephalosporin ceftazidime (D. Zincke, personal communication). β-Lactamase quantification showed that PAOΔ*ampR* produced significantly lower amounts in response to β-lactam stress compared to PAO1 (PAO1: 11.27 mU vs. PAOΔ*ampR*: 6.5 mU, *p* value 0.0003; Fig. 1B), which is in agreement with the E-test data. The loss of induction was recovered by expressing *ampR* from a low-copy plasmid (PAOΔ*ampR*: 6.5 mU vs. PAOΔ*ampR* (pAmpR): 11.35 mU, *p* value 0.004; Fig. 1B). These data clearly reiterate the role of AmpR in β-lactam resistance in *P. aeruginosa* as previously described [21]. The PAOΔ*ampR* strain was used for all further assays.

### B. Loss of ampR affects ability of PAO1 to kill *C. elegans*

**Figure 2:**
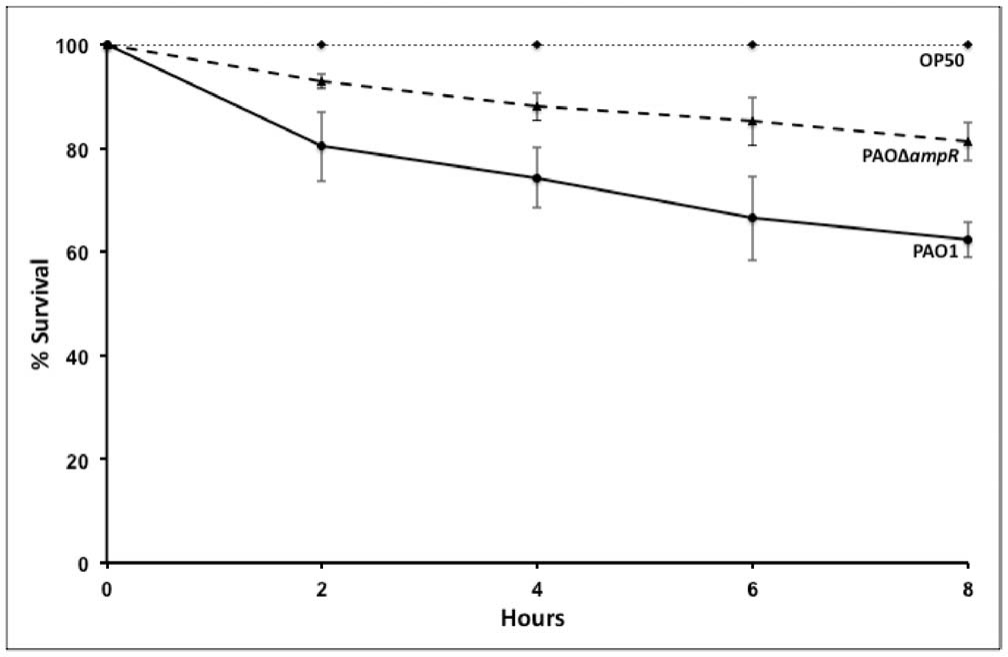
Effect of *ampR* deletion on pathogenicity to *C. elegans*. The fast killing assay was used to test the effect of loss of *ampR* on the *C. elegans* killing ability of PAO1. *p*-value<0.05 at all time points.

The importance of *ampR* in virulence was determined in a *C. elegans* model, as reported previously [29], [30]. Using the fast killing (paralytic) assay, we monitored the ability of PAO1 and its isogenic *ampR* mutant, PAOΔ*ampR* to kill *C. elegans* over eight hours. PAOΔ*ampR* showed reduced pathogenicity, killing only 15% of the nematodes compared to the 38% killed by the wild-type PAO1 at the end of the study period (*p* value<0.05 at all time points; Fig. 2). The results indicate that a functional AmpR is required for full pathogenicity of *P. aeruginosa* in the nematode model. To characterize the full extent of AmpR-mediated regulation of *P. aeruginosa* pathogenesis, we analyzed PAOΔ*ampR* further.

### C. AmpR regulates numerous genes in *P. aeruginosa*

**Figure 3:**
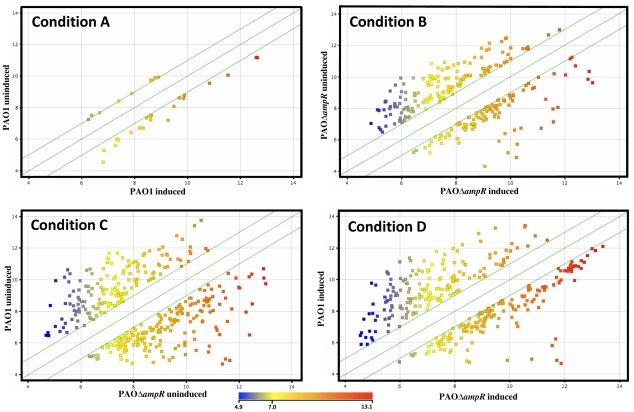
Scatter plots of significantly regulated genes. Only genes that showed significant (*p*≤0.01) differential regulation under the various conditions are depicted as colored squares. The colors represent the extent of gene expression from low (blue) to high (red) in either condition, as depicted in the color scale. The two outer green diagonal lines in each plot represent the two-fold cutoff. Each sub-plot depicts the differential gene expression between two strains/conditions (shown along the plot axes): Condition A- PAO1 uninduced vs. PAO1 induced; Condition B- PAOΔ*ampR* uninduced vs. PAOΔ*ampR* induced; Condition C- PAO1 uninduced vs. PAOΔ*ampR* uninduced; Condition D- PAO1 induced vs. PAOΔ*ampR* induced.

Using DNA microarrays, we compared the expression profiles of PAO1 and PAOΔ*ampR*, without (uninduced) and with (induced) sub-MIC β-lactam stress to identify genes that are regulated under the different conditions. Pair-wise comparisons of the datasets of significantly differentially regulated genes (*p* value≤0.01, ≥two-fold) either with or without sub-MIC β-lactam stress led to the identification of 32 genes (PAO1 uninduced vs. PAO1 induced; Condition A), 258 genes (PAOΔ*ampR* uninduced vs. PAOΔ*ampR* induced; Condition B), 345 genes (PAO1 uninduced vs. PAOΔ*ampR* uninduced; Condition C) and 338 genes (PAO1 induced vs. PAOΔ*ampR* induced; Condition D) (Fig. 3). As seen in Figure 3, the expression of 345 genes is altered in the absence of AmpR (Condition C), clearly indicating that AmpR influences the expression of numerous genes in *P. aeruginosa*.

Quantitative real-time PCR (qPCR) was used to verify the microarray results. Genes for the verification analysis were selected across the spectrum of regulation, based on the raw microarray reads after normalization, including both up and downregulated genes. Twelve genes were selected for initial qPCR analysis, six each from the up and downregulated sets, using *clpX* (*PA1802*) as the reference control gene since *clpX* expression did not change in our microarray data between the strains and conditions tested. qPCR data showed the same trend of either up- or downregulation of the genes as in the microarray, validating our microarray observations, notwithstanding the variations expected due to differences in the sensitivity of the two assays (Table 1).

**Table 1:** Microarray vs. qPCR.

Locus ID/Gene	Fold change	
	Microarray	qPCR
PA0610/*prtN*	12	6
PA0762/*algU*	5	3
PA3602	5	10
PA2493/*mexE*	108	8089
PA4121	7	2
PA1708/*popB*	−8	−4
PA2193/*hcnA*	−7	−3
PA1078/*flgC*	−5	−2
PA2069	−38	−93
PA2331	−7	−40

Confirmation of microarray data was performed by qPCR, using RNA isolated from cells as explained in the text. Data shown is gene expression in penicillin non-treated PAOΔ*ampR* cells, normalized to expression in PAO1.

### D. AmpR regulates genes both in the absence and presence of β-lactam stress

**Figure 4:**
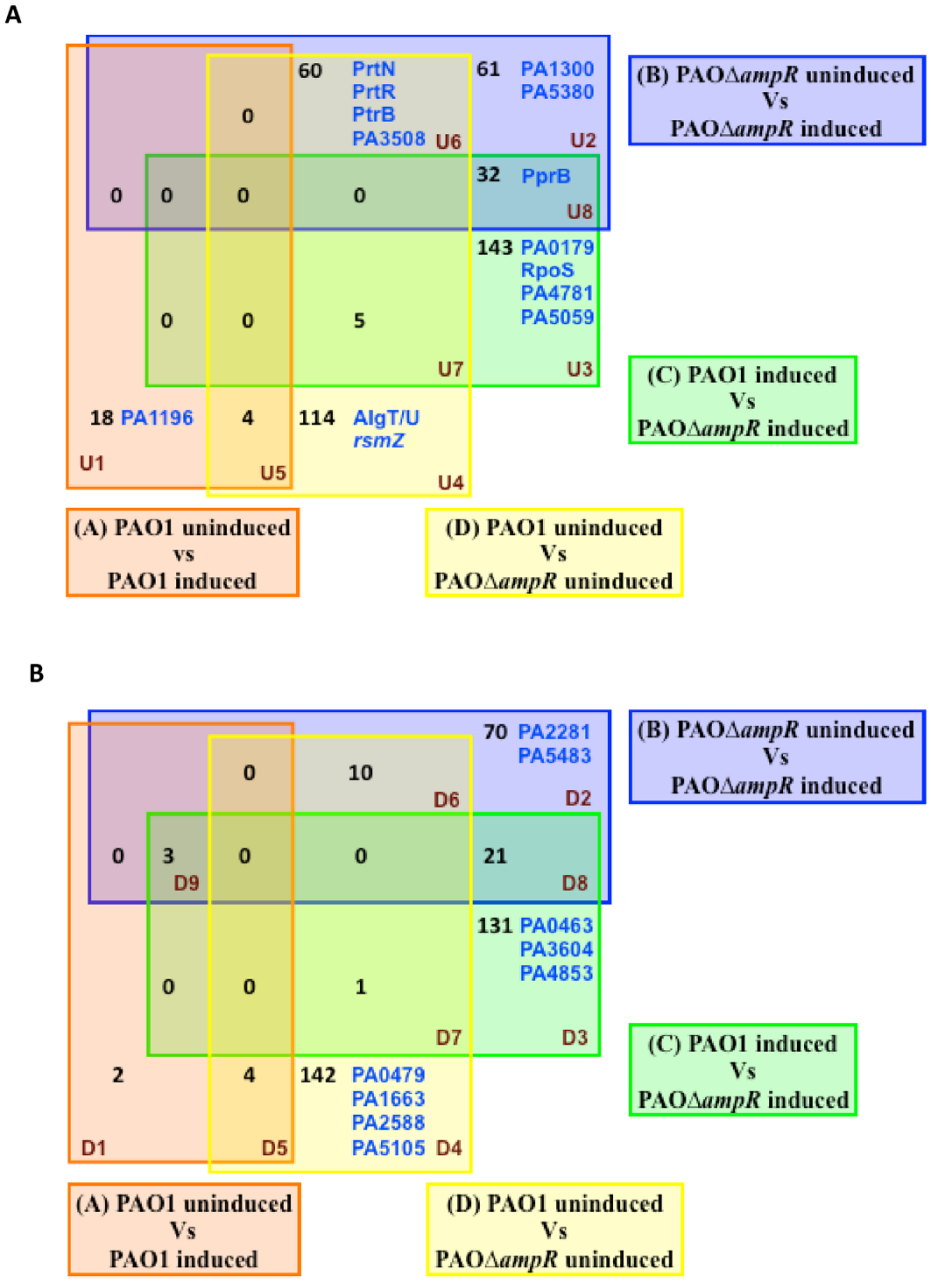
Venn diagram of the differentially regulated genes. Distribution of significantly (*p*≤0.01) differentially regulated (>2-fold) genes in PAO1 and PAOΔ*ampR* with (induced) and without (uninduced) β-lactam treatment showing upregulated (Fig. 4A) and downregulated (Fig. 4B) genes. The transcriptional regulators, sigma factors and small RNAs in each group are identified either by their gene names or PA numbers. Annotations are from the *Pseudomonas* Genome database [31].

Subsets of genes that are differentially regulated either due to loss of *ampR* or due to β-lactam antibiotic exposure or both (Fig. 3) could potentially be regulated under more than one condition and this overlap would be misinterpreted in the total number of genes regulated in each condition. To address this issue, the 973 differentially regulated genes (*p*≤0.01, FC≥2-fold) in the four pair-wise comparisons (Conditions A–D in Fig. 3) were plotted in 4-way Venn diagrams and separated into upregulated (Fig. 4A) and downregulated (Fig. 4B) genes.

From these two Venn diagrams, genes that were dependent exclusively on either AmpR or β-lactams or on both were identified. Comparison of Conditions A and B yield genes unique to each condition (U1, D1 and U2, D2 respectively in Fig. 4A–4B). There are 18 upregulated genes (U1 in Fig. 4A) that are unique to Condition A (response of PAO1 to β-lactam stress). This includes AmpR-dependent genes (there is a functional AmpR in these strains that helps in the response to β-lactams) and AmpR-independent genes (some of these genes may be regulated in an AmpR-independent manner). In contrast, in Condition B (response of PAOΔ*ampR* to β-lactam stress), 61 genes are upregulated in response to antibiotics (U2 in Fig. 4A). AmpR may negatively regulate these genes, since their expression is enhanced in the absence of AmpR, or their expression is AmpR-independent. The observation that loss of *ampR* leads altered gene expression in PAO1 in response to sub-MIC β-lactam stress (similar to that seen in the absence of β-lactam stress) further hints at a global regulatory role for AmpR in *P. aeruginosa*.

Of the 18 upregulated genes in U1 (PAO1 induced), there could also be a subset of genes that are AmpR- and β-lactam dependent due to a functional AmpR in PAO1. In order to identify and separate exclusively β-lactam regulated genes, we compared these 18 upregulated genes to see if they were downregulated in Condition B (in D2) and identified *PA0465* (*creD*), *PA0466* (hypothetical) and *PA3889* (hypothetical). So, of the 18 genes in U1, only these three genes are specifically AmpR-regulated. Using a similar logic (for key, see Table S1), we identified β-lactam dependent genes (Table S2A, Table S2B) to fall in regions U6, D6, U7, D7, U8 and D8 in addition to U1 and D1 of the Venn diagram (Fig. 4A and 4B). The genes that were regulated exclusively in response to β-lactam antibiotics include *mrcB*, a penicillin-binding protein 1 (PBP1) that is involved in peptidoglycan synthesis and is upregulated 3.5-fold (corrected *p*-value 3.71E-03). Genes of putative RND efflux and ABC transporters are also upregulated (Table S2A), as are genes involved in flagellar biosynthesis and the *sox* operon (*PA5416*–*PA5419*; involved in amino acid metabolism). Genes that are downregulated in response to β-lactam exposure include 10 membrane proteins suggesting adaptation to stress, in addition to genes of the *phz2* phenazine biosynthetic operon.

The genes that are AmpR-dependent (U2, D2, U4 and D4: 387 genes) and AmpR-β-lactam dependent (U3, D3, U5 and D5: 282 genes) (Fig. 4A and 4B) are of interest to us, since AmpR influences both these gene sets. To further eliminate β-lactam specific genes, we compared the AmpR-dependent and AmpR-β-lactam dependent gene lists between themselves as well as to the 206 β-lactam-specific genes (Table S2A). This led to the identification of 520 genes whose expression is influenced by AmpR, of which 313 are AmpR-dependent (Table S3A, Table S3B) and a further 207 that are AmpR-β-lactam dependent (Table S4A, Table S4B).

### E. Functional categorization of AmpR-regulated genes

**Figure 5:**
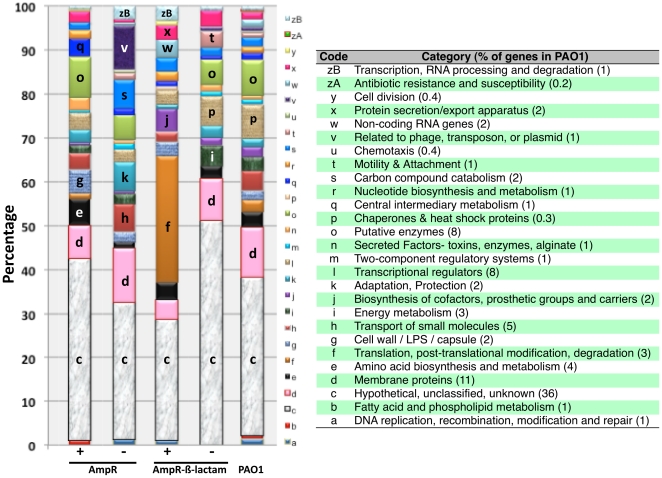
Enrichment of functional categories. Functional categorization of the AmpR-dependent and AmpR-β-lactam dependent genes was performed to identify enrichment of specific classes of genes relative to their distribution in PAO1. ‘+’ and ‘−’ refers to upregulation and downregulation of genes, respectively in either an AmpR-dependent or AmpR-β-lactam-dependent manner. The most differentially regulated categories are labeled in the figure using the corresponding code, mentioned in the figure table. Functional categories, and their codes and percentages in PAO1 are from the *Pseudomonas* Genome database [31].

Functional categorization of the 520 AmpR-regulated genes was then performed and compared to the distribution in the *P. aeruginosa* PAO1 genome from the *Pseudomonas* Genome database [31] to look for enrichment of specific classes of genes in the different conditions (Fig. 5). Category N genes (secreted factors, toxins, enzymes, alginate) were found to be downregulated in an AmpR-dependent manner and this correlated with decreased production of some secreted virulence factors (elaborated in the following sections). Similarly, genes in category G (cell wall, LPS, capsule) were downregulated, indicating that AmpR positively regulates these genes. Moreover, metabolic genes (amino acid biosynthesis - category E; central intermediary metabolism - category Q) show marginal downregulation in the *ampR* mutant. Genes involved in carbon compound catabolism (category S), adaptation and protection (category K), and those related to phage/transposon/plasmid (category V) were upregulated in the PAOΔ*ampR*, indicating negative AmpR regulation in an AmpR-dependent manner (Fig. 5). Interestingly, genes encoding transcriptional regulators (category L) show differential regulation indicating that AmpR could potentially regulate other genes indirectly via intermediate transcriptional regulators.

In the presence of β-lactam stress, loss of *ampR* leads to downregulation of membrane proteins (category D), putative enzymes (category O) and those involved in translation (category F), while motility and attachment genes (category T) were upregulated (Fig. 5). In addition to membrane proteins (category D), the most differential regulation across all conditions was of the hypothetical proteins (category C), but until the functions of these proteins are determined, no significant conclusions can be drawn.

### F. AmpR binding site analysis

**Figure 6:**
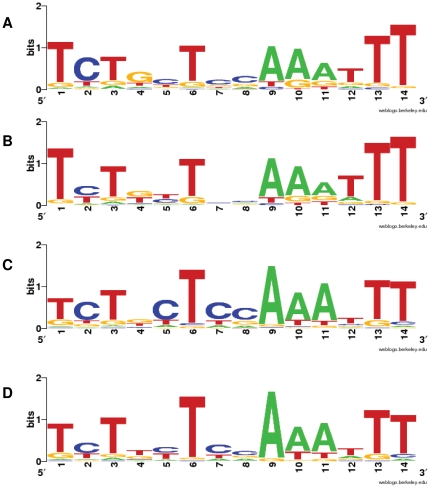
AmpR binding site analysis. The putative AmpR binding site was refined using IEM algorithm and used to search the upstream promoter elements of AmpR-regulated and AmpR-β-lactam regulated genes (listed in Tables S3, S4) in PAO1 and other *P. aeruginosa* strains. DOOR database [142], [143] was used to identify the operons. The RSAT tool [137] was used to identify PAO1 promoter sequences containing the identified AmpR- and AmpR-β-lactam-regulated genes. The output is represented using WebLogo [136] for the AmpR-dependent genes from IEM (A) and RSA (B), and AmpR-β-lactam dependent genes from IEM (C) and RSA (D).

In an attempt to identify the genes that are directly regulated by AmpR, a bioinformatics approach was adopted. IEM and RSA analyses were performed to identify, refine and scan the *P. aeruginosa* genomes as well as the AmpR-regulated genes from the microarray data for potential AmpR-binding sites. The consensus sequences derived from the AmpR- and AmpR-β-lactam dependent genes are similar, yet distinct (Fig. 6A and 6C). Using these consensus sequences to search the upstream regulatory regions of the PAO1 genome led to the identification of the AmpR-dependent element in the upstream region of 244 genes and the AmpR-β-lactam-dependent element in the upstream region of 207 genes. The motifs derived from the IEM and RSA analysis for both the AmpR-dependent and AmpR-β-lactam-dependent genes are almost identical (Fig. 6). Of the genes identified in the microarray analysis, only 11.9% of AmpR-dependent and 14.5% of AmpR-β-lactam-dependent upstream regulatory regions were identified by IEM or RSA analysis as having an AmpR binding site. This suggests that perhaps AmpR is exerting its effect by either directly or indirectly altering expression of a global transcription regulator. Studies aimed at identifying the direct targets of AmpR are needed to identify genes that are directly regulated by AmpR.

### G. Regulation of the amp genes by AmpR

**Figure 7:**
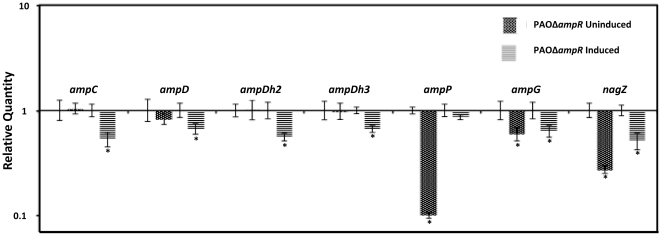
Expression of the *amp* genes in PAOΔ*ampR*. RNA was isolated from cells exposed to β-lactam antibiotic, reverse transcribed to cDNA and tested in triplicate by qPCR with gene-specific primers, as described in the text. The expression of *ampC* (β-lactamase), *ampD*, *ampDh2*, and *ampDh3* (amidases), *ampG* and *ampP* (permeases), and *nagZ* (glycoside hydrolase) were tested in the *ampR* mutant relative to PAO1. Values have been normalized to expression in PAO1 under the same conditions (log_10_ RQ = 1) and bars above or below the line represent up- and down-regulation, respectively. * *p* value<0.05.

β-Lactam resistance in *P. aeruginosa* is mediated, in part, by the *amp* genes that are also tied in with the cell wall recycling process [32]. The genes involved in this process include the regulator AmpR [21], [33], the chromosomal β-lactamase AmpC [11], [12], [28], [34], the permeases AmpG and AmpP [15], [35] and the amidases AmpD, AmpDh2 and AmpDh3 [36], [37]. In addition, the hydrolase NagZ also plays a role in β-lactam resistance [38], [39]. Since AmpR is known to positively regulate AmpC expression, we hypothesized that AmpR also regulates the other *amp* genes. qPCR analysis revealed downregulation of the *amp* genes in PAOΔ*ampR*, normalized to expression in PAO1 (Fig. 7), indicating a positive regulatory role for AmpR in the expression of *ampG* (RQ - uninduced: 0.59±0.01, *p*-value 0.006; induced: 0.63±0.01, *p*-value 0.04), *ampP* (RQ - uninduced: 0.1±0.01, *p*-value 0.004; induced- 0.86±0.01, *p*-value NS), *ampD* (RQ - uninduced: 0.82±0.01, *p*-value NS; induced 0.67±0.01, *p*-value 0.04), *ampDh2* (RQ - uninduced: 1.01±0.05, *p*-value NS; induced- 0.56±0.01, *p*-value 0.002), *ampDh3* (RQ - uninduced: 0.98±0.007, *p*-value NS; induced: 0.66±0.04, *p*-value 0.02) and *nagZ* (RQ - uninduced: 0.27±0.06, *p*-value 0.002; induced: 0.51±0.01, *p*-value 0.0002). Specifically, when the cells are exposed to β-lactams, they need amidase activity to help in the peptidoglycan recycling process. This is reflected in upregulation of the amidases, (AmpD, AmpDh2, and AmpDh3) and AmpC only when the cells are exposed to β-lactams. Simultaneously, AmpG, which functions to transport the degraded peptidoglycan material into the cytoplasm [15] is upregulated by AmpR (downregulated in PAOΔ*ampR*) in an inducer-independent manner. This shows that AmpR upregulates the amidases and AmpC β-lactamase in response to β-lactams while upregulating AmpG (Fig. 7), and agrees with the proposed model for peptidoglycan recycling in *P. aeruginosa* [15]. The data, thus, demonstrates the central role of AmpR in influencing expression of the cell wall recycling/AmpC-mediated β-lactam resistance machinery in *P. aeruginosa*.

### H. AmpR regulates the expression of antibiotic resistance and virulence systems

It has previously been shown that AmpR regulates the expression of genes related to QS and protease production [40]. Microarray analyses from this study show that AmpR affects the expression of multiple virulence systems in *P. aeruginosa*, as explained further in this section.

#### Resistance-Nodulation-Division (RND) efflux systems

RND transporters are tripartite pumps present in Gram-negative bacteria that are involved in the efflux of antibiotics and several other compounds decreasing cytoplasmic retention and thus conferring resistance [41]. All of the *P. aeruginosa* strains sequenced so far carry 12 known and putative RND efflux pumps, suggesting that the efflux pumps are an integral part of the *P. aeruginosa* genome [42], [43]. The MexEF-OprN efflux system that is primarily concerned with resistance to fluoroquinolones, chloramphenicol and trimethoprim [44] was significantly upregulated in PAOΔ*ampR*. The genes *mexE* (seven-fold), *mexF* (89-fold) and *oprN* (103-fold) are overexpressed in the *ampR* mutant in the absence of antibiotic stress in microarray studies (Table S3A) and overexpression of the first gene of the operon, *mexE*, was confirmed by qPCR (Table 1). MexT, an activator of this efflux system [44], [45], is not significantly differentially regulated (1-fold) in the microarray studies, but is upregulated in an inducer-independent manner when tested by qPCR (RQ: uninduced 7.5±0.11, *p*-value 0.02; induced 7.0±0.25, *p*-value 0.02). However, the negative regulator MexS shows no differential regulation either in microarray or qPCR analysis (data not shown). Carbapenems use the outer membrane porin OprD to gain entry into the cell [46] and MexT negatively regulates this porin both at the transcriptional and post-transcriptional level [26], [47], [48]. Indeed, qPCR analysis shows that with upregulation of *mexT*, there is a downregulation of *oprD* expression in the *ampR* mutant compared to PAO1 (RQ: uninduced 0.31±0.004, *p*-value 0.001; induced 0.08±0.000002, *p*-value<0.0001). Surprisingly, OprD downregulation did not lead to increased resistance to imipenem and meropenem (Fig. 1A). In addition, we did not see differential regulation of the other known MexT regulator, MvaT [49] in our microarray analysis.

To further investigate whether upregulation of this pump translates into a resistance phenotype, we determined the MICs for MexEF-OprN substrate antibiotics by the standard broth microdilution method [50]. PAOΔ*ampR* showed enhanced resistance to four of the antibiotics tested when compared to the resistance profile of wild-type PAO1, correlating microarray and qPCR data with the phenotype (Table 2). This suggests that AmpR is involved in resistance to β-lactam antibiotics by regulating AmpC as shown earlier, and non-β-lactam antibiotics via the MexEF-OprN efflux pump. Analysis of the promoter regions of the genes of this pump and their regulators (MexS and MexT) using the putative AmpR-binding site [51] as a query sequence did not reveal signs of AmpR binding, suggesting indirect regulation by AmpR.

**Table 2:** *ampR* deletion affects susceptibility to MexEF-OprN substrates.

Antibiotic	MIC (µg/ml)	
	PAO1	PAOΔ*ampR*
**Ofloxacin**	4	32
**Ciprofloxacin**	0.25	2
**Chloramphenicol**	128	512
**Trimethoprim**	200	>200

The minimum inhibitory concentrations (in µg/ml) of MexEF-OprN substrate antibiotics to PAO1 and PAOΔ*ampR* were determined by broth microdilution method (see text for details).

MexAB-OprM was the first RND-type efflux pump to be reported in *P. aeruginosa* and has very broad substrate specificity including β-lactam antibiotics and non-antibiotic substrates [52]. In fact, it has been implicated to play a more significant role in resistance to β-lactam antibiotics than β-lactamases [53], [54]. Using the putative *P. aeruginosa* AmpR binding site [51], we identified a potential AmpR binding site upstream of the MexR repressor of this pump in the MexR-MexA intergenic region (5′ AAGCCTGCAAATGT 3′) indicating possible regulation of this pump by AmpR. qPCR analysis of *mexR* expression revealed downregulation of this gene in PAOΔ*ampR* compared to PAO1 (RQ: uninduced 0.46±0.006, *p*-value 0.002; induced 0.4±0.007, *p*-value 0.01). It is thus interesting to note that AmpR not only positively regulates AmpC β-lactamase but potentially also MexAB-OprM, two different mechanisms of resistance to β-lactams. The MexAB-OprM efflux can also pump out fluoroquinolones [26] and the enhanced quinolone resistance of PAOΔ*ampR* seen in the MIC studies is potentially due to a combined effect of upregulation of the MexEF-OprN and the MexAB-OprM efflux pumps. In addition, AmpR negatively regulates a two-gene putative RND efflux operon *PA1435*–*PA1436* (nine-fold and four-fold respectively in microarray) that codes for a membrane fusion protein and efflux transporter, respectively. Potential AmpR regulation of the MexGHI-OpmD efflux pump is discussed in the QS section.

#### QS-regulated virulence factors

Many of *P. aeruginosa* virulence factors are QS-regulated and form a critical component of pathogenesis [55], [56]. In our previous meta-analysis of published *P. aeruginosa* transcriptomes, we identified differentially regulated sets of system-specific and condition-specific genes, including QS-regulated genes [57]. Using this as our knowledge base, the *ampR* microarray profile was compared to identify differentially regulated QS-specific genes. The microarray data shows that AmpR influences expression of many QS-regulated genes (Table S5).

To further verify AmpR-mediated regulation of QS virulence factors, we quantified the production of pyocyanin, LasA protease, and LasB elastase. Pyocyanin is a redox active exotoxin pigment that contributes to lung pathophysiology of chronic *P. aeruginosa* infections [58] and interferes with multiple host cellular functions [59]. Genes in the locus of the *phz1* operon that is involved in QS-regulated phenazine synthesis, including *phzA1* (*PA4210*, 4-fold down), *phzB1* (*PA4211*, 21-fold down), *phzS* (*PA4217*, 28-fold down), *phzM* (*PA4209*, 4-fold down), and the MexGHI-OpmD efflux pump (*PA4205*–*PA4208*, 11–30-fold down) that plays a role in pumping out the pigments [60], show decreased expression in PAOΔ*ampR*. In agreement with this data, there was a statistically significant (*p*-value<0.0001) reduction in pyocyanin production by the *ampR* mutant, compared to PAO1 and this effect was independent of β-lactam stress on the cells (Table 3). The data indicates that AmpR influences pyocyanin production which is in agreement with the *C. elegans* killing data (Fig. 2), since phenazines are major players in *C. elegans* mortality in the fast-killing assay [30].

**Table 3:** Effect of deletion of *ampR* on QS-regulated virulence phenotypes.

Strain	Pyocyanin production^a^		LasA activity^b^		LasB activity^c^	
	Uninduced	Induced^d^	Uninduced	Induced^d^	Uninduced	Induced^d^
**PAO1**	22.31±0.18	24.30±1.10	0.37±0.005	0.39±0.004	1.65±0.25	1.54±0.15
**PAOΔ** ***ampR***	2.29±0.11^e^	5.74±0.56^f^	0.26±0.002	0.19±0.002^g^	0.5±0.03^h^	0.57±0.08^i^

a Pyocyanin concentrations were expressed as micrograms of pyocyanin produced per microgram of protein.

b LasA activity was expressed as change in OD600 per hour per microgram of protein.

c LasB elastase activity was expressed as change in OD495 per microgram of protein.

d Induction was carried out with 100 µg/ml of penicillinG.

e *p*-value<0.0001 when comparing PAO1 and PAOΔ*ampR*.

f *p-*value<0.0001 when comparing PAO1 and PAOΔ*ampR*.

g,h *p-*value of 0.02 when comparing PAO1 and PAOΔ*ampR*.

i *p-value* of 0.01 when comparing PAO1 and PAOΔ*ampR*.

Elastases (pseudolysins) are highly toxic zinc metalloproteases that play a critical role in immunomodulation [61], [62], host tissue damage aiding invasion [63] and biofilm formation [64]. The LasB elastase production was also severely affected due to the loss of *ampR* (*p*-value≤0.02) in an inducer-independent manner (Table 3). Along with LasB, a zinc metalloendopeptidase, LasA plays a major role in *P. aeruginosa*-induced keratitis [65]. Reduction in LasA protease production, however, was significantly lower (*p*-value<0.05) in PAOΔ*ampR* only when the strains were exposed to sub-MIC β-lactam stress (Table 3), and this is in agreement with data from microarray (8-fold downregulated) and qPCR (RQ: uninduced- NS; induced 0.21±0.07, *p*-value<0.0001) analysis.

Microarray data shows that loss of *ampR* also affects other QS-regulated virulence genes, such as the *hcn* operon *PA2193–PA2195* (5 to 7-fold downregulated) that is involved in the production of hydrogen cyanide. Cyanide toxicity is the primary mode of fast killing of *C. elegans* by *P. aeruginosa* PAO1 [66]. The downregulation of the *hcn* operon concurs with reduced killing by PAOΔ*ampR*. The expression of the galactophilic lectin *lecA* (*PA2570*) is also downregulated in PAOΔ*ampR* (RQ: uninduced- 0.22±0.0005, *p*-value 0.0001; induced: 0.16±0.003, *p*-value 0.0005) indicating positive AmpR regulation. LecA facilitates bacterial entry into host cells by aiding in adhesion to endothelia and epithelia [67] and is involved in biofilm formation [68]. It has previously been shown that *lecA* expression is regulated by the sigma factor RpoS [69] and by the QS regulator RhlR [69]. Thus, the effect of AmpR on *lecA* expression could be mediated indirectly via RpoS and/or RhlR. As predicted, RpoS was downregulated (RQ: uninduced- 0.55±0.08, *p*-value 0.01; induced 0.51±0.02, *p*-value 0.0003), indicating AmpR positive regulation in a β-lactam-independent manner. Also, since we see downregulation of multiple QS phenotypes, RhlR is also potentially involved in AmpR-mediated *lecA* regulation.

QS activates the operon *PA2327*–*PA2331* [70] coding for a probable ABC transporter. Wolfgang *et al.* found this operon to be repressed when *P. aeruginosa* was grown in CF respiratory liquid containing media [71]. Genes of the operon are significantly upregulated (6- to 18-fold) in PAOΔ*ampR* indicating that AmpR negatively regulates this operon and further connects AmpR to QS regulation, adding another regulatory player in this complex regulatory network.

#### Biofilms

**Figure 8:**
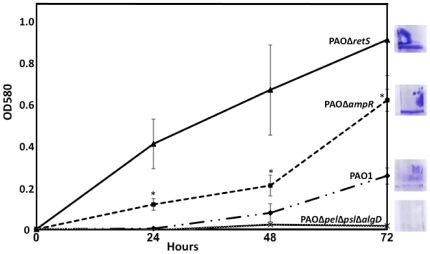
AmpR affects biofilm formation. AmpR regulates many genes involved in biofilm formation (Table S6) that is reflected in the tube biofilm assay monitored for 72 hours. The strains tested were wild type PAO1, PAOΔ*ampR*, PAOΔ*retS* (positive control), and PAOΔ*pel*Δ*psl*Δ*algD* (negative control). The inset, taken at 72 hours, demonstrates the superior biofilm formation capacity of PAOΔ*ampR* compared to PAO1. *p*-values comparing PAOΔ*ampR* with PAO1: 24 hrs- 0.002; 48 hrs- 0.03; 72 hrs- 0.007.

Successful biofilm formation is dependent on nutrient availability, motility and QS [72]. Comparison of gene expression profiles from this study with the biofilm-specific gene list generated from our previous analysis of *P. aeruginosa* transcriptomes [57] revealed the differential regulation of many biofilm genes in PAOΔ*ampR* (Table S6). This suggests a role for AmpR in biofilm regulation, either directly or indirectly. Testing the tube biofilm-forming ability of the strains revealed that PAOΔ*ampR* formed better biofilms, compared to PAO1 (Fig. 8). The difference was significant at all time points tested (*p*-value≤0.03) over a period of 72 hours indicating that AmpR is a negative regulator of biofilm formation.

Microarray data also revealed upregulation of *PA4651* (encoding a probable pili assembly protein; 11.6-fold), and PA4306 (coding for Flp, Type IVb pilin, 36-fold) in PAOΔ*ampR*, in the absence of antibiotics. Since type IV pili-mediated twitching and flagella-mediated swimming motilities are known proponents for biofilm formation at different stages [73], we tested for these phenotypes with PAO1 and PAOΔ*ampR*. In the absence of β-lactam antibiotic stress, the PAOΔ*ampR* strain demonstrated enhanced twitching ability (PAOΔ*ampR*: 15.1 mm±1.1 mm; PAO1: 2 mm±0.1 mm; *p* value<0.0001) potentially explaining its ability to form better biofilms. Moreover, unlike in PAO1, under sub-MIC β-lactam stress, PAOΔ*ampR* showed a marginal but statistically significant increase in twitching zones (uninduced: 15.1 mm±1.1 mm; induced: 16.5 mm±0.8 mm; *p*-value 0.04). This observation is in agreement with the enrichment of the gene set for the ‘motility and attachment’ functional category under β-lactam stress in PAOΔ*ampR* (see section E above). There was, however, no difference in the swimming motility of the strains (data not shown), although *fleR* of the FleSR two-component system that is involved in the flagella biosynthesis regulatory pathway [74] was differentially regulated in the *ampR* mutant (RQ: uninduced- 0.45±0.1, *p*-value 0.0007; induced- 0.32±0.01, *p*-value 0.0006).

The Pel polysaccharide is a glucose-rich exopolymer, encoded by the *pel* operon (*PA3058–PA3064*) that along with the mannose-rich Psl polysaccharide plays a major role in pellicle formation [75], [76]. PAOΔ*ampR* forms darker red colonies on Tryptone-Congo red agar plates compared to PAO1 (data not shown) signifying higher Pel production [75]. This is consistent with the observation that the *ampR* mutant produces better biofilms. Collectively, these data suggest that AmpR negatively influences biofilm formation in *P. aeruginosa* either directly or indirectly.

Recently, a novel efflux pump that confers antibiotic resistance in *P. aeruginosa* biofilms has been identified [77]. Deletion of the operon (*PA1874*–*1877*) encoding this pump in PAO1 enhances sensitivity to gentamycin, tobramycin and ciprofloxacin, especially in biofilm cells. Genes in this operon are downregulated four-fold in the *ampR* mutant strain. This observation, though not verified separately, suggests that AmpR positively regulates this operon, thus possibly contributing to non-β-lactam antibiotic resistance in biofilms.

#### Secretory systems

**Figure 9:**
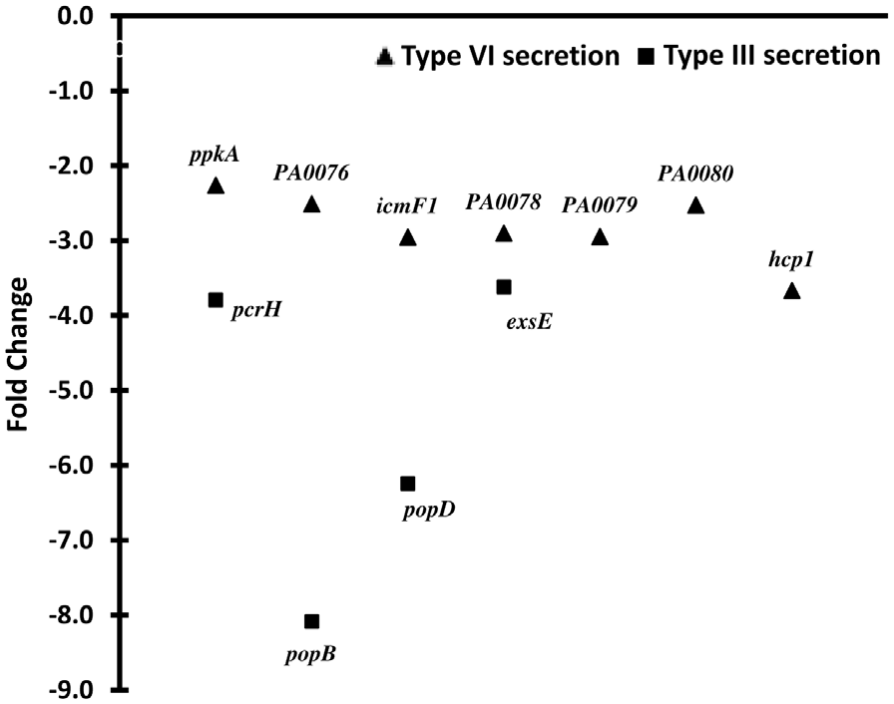
Regulation of secretion genes by AmpR. Fold changes (FC) as determined by microarray experiments in the absence of β-lactam stress of the Type VI secretion (▴, HSI-I) and Type III secretion (▪) genes in the *ampR* mutant strain, normalized to expression in the wild-type strain PAO1. Gene annotations are from the *Pseudomonas* Genome database [31].

The type III secretion system (T3SS) is essential for *P. aeruginosa* not only for contact-dependent toxin delivery to host cells but also for phagocyte evasion [78], [79]. Using a burn mouse model, it was shown that loss of T3SS results in reduced virulence [80]. The genes encoding the regulatory and structural components of the T3SS in *P. aeruginosa* are concentrated in one locus (*PA1690*–*PA1725*) whereas the effectors and their chaperones are scattered in the genome [31]. Deletion of *ampR* from PAO1 led to the downregulation of a few T3SS genes in the regulatory-structural gene cluster (Fig. 9) indicating positive AmpR-regulation. These include the genes encoding the regulators PcrH (involved in regulating ExoS synthesis) and ExsE (a secreted regulator of the ExsCED regulatory cascade), and the translocator proteins PopB and PopD. qPCR confirmation of the microarray findings for the T3SS genes was done using RNA isolated from Ca^2+^ depleted inducing media (MinS-NTA), since Ca^2+^ is a known inhibitor of T3SS [81]. Since *pcrH*, *popB* and *popD* are the last three genes of a 12-gene operon, we tested expression of only *pcrH* by qPCR, which showed a downregulation in PAOΔ*ampR* compared to PAO1 (RQ: uninduced- 0.71±0.06, *p*-value 0.02; induced- 0.88±0.03, *p*-value 0.003). Similarly, *exsE*, the second gene of the *exsCEB* operon was also downregulated in PAOΔ*ampR* (RQ: uninduced- 0.57±0.01, *p*-value 0.001; induced- 0.69±0.18, *p*-value NS) indicating positive regulation of these genes by AmpR.

Transcriptional regulation of T3SS in *P. aeruginosa* is a complex process and involves multiple tiers of regulation [81]. One of the mechanisms of control involves a small RNA-binding protein RsmA and non-coding small RNAs (sRNA), *rsmY* and *rsmZ*. Sequestration of RsmA by the sRNAs inhibits its activity. RsmA has an extensive virulence regulon that is tied in with the GacSA two-component system regulatory cascade [82], [83]. In PAOΔ*ampR*, *rsmZ* is downregulated (RQ: uninduced- 0.21±0.03, *p*-value 0.01; induced- 0.78±0.09, *p*-value NS) and corresponds with an upregulation of RsmA (in microarray, uninduced - 2.3 fold; corrected *p*-value 0.009; induced- NS). Some of the T3SS genes are, however, downregulated in the *ampR* mutant (Fig. 9). RsmA is a positive regulator of T3SS. This suggests that the effect of AmpR on T3SS is probably not via RsmA, or involves an additional step of post-transcriptional regulation. PtrB represses T3SS gene expression [84] and since AmpR regulates PtrB expression (see Section H below), AmpR regulation of T3SS is potentially via PtrB.

*P. aeruginosa* T6SS is involved in chronic CF infections [85]. RsmA also negatively regulates genes of the Type 6 secretion system (T6SS), particularly of the HSI-I system [83]. Thus, downregulation of genes of the T6SS in PAOΔ*ampR* (Fig. 9) is possibly through an indirect effect of AmpR on RsmA.

### I. AmpR regulates genes found in regions of genome plasticity (RGP)

Comparative analysis of five *P. aeruginosa* chromosomes identified RGPs that are strain-specific [43]. These are genome segments that can be acquired by horizontal gene transfer, bacteriophages or transposons [43]. We wanted to determine whether an endogenous transcriptional regulator, such as AmpR, could regulate expression of genes acquired by the strain, such as RGP genes. Microarray analysis revealed that in the PAOΔ*ampR* mutant, 31 RGP genes are regulated in an AmpR-dependent manner and an additional eight RGP genes under sub-MIC β-lactam stress (Table S7).

Most of the RGP03 (*PA0612*–*PA0628*) and RGP04 (*PA0641*–*PA0648*) ORFs belong to functional category V (related to phage, transposon and plasmid) and code for the two classes of high molecular weight pyocins, types R and F. Pyocins R and F are related to bacteriophage tails and kill susceptible cells thus conferring a survival advantage on the producing strain [86]. Thirty-two percent of the RGP genes regulated in an AmpR-dependent manner are clustered and localized to RGP03. The expression of 10 RGP03 genes and two RGP04 genes (59% and 20% of genes in RGP03 and RGP04, respectively) are significantly upregulated (6–8-fold) in PAOΔ*ampR* as seen in microarray data (Table S7). This suggests that AmpR is involved in negatively regulating these genes, either directly or indirectly.

PrtN (PA0610), and the product of the first gene in RGP03, *ptrB* (*PA0612*), is a positive regulator of pyocin production, both of which are repressed by PrtR (PA0611) [87]. Upon DNA damage, RecA (PA3617) degrades PrtR, thus inducing pyocin production [87]. In PAOΔ*ampR* subjected to sub-MIC β-lactam stress compared to PAO1, *prtN* (12-fold), *recA* (3-fold) and *ptrB* (20-fold) are upregulated, while the negative regulator PrtR is downregulated (qPCR relative quantity: uninduced- 0.88±0.4, *p*-value *p*-value 0.004; induced- 0.76±0.109 *p*-value 0.0002) implying that AmpR is a negative regulator of pyocin production.

### J. AmpR regulates other transcriptional regulators

The AmpR-regulation of hundreds of genes (Fig. 4A and 4B) could be by direct binding to the promoters or in a subset of genes, indirectly through intermediate transcriptional regulators, sigma factors or regulatory RNAs. In a preliminary attempt to further elucidate the AmpR regulon, we looked at the transcriptional regulators and sigma factors that are AmpR-regulated. Of the 430 transcriptional regulators in *P. aeruginosa* PAO1 [31], 19 met the selection criteria in our microarray analysis (see materials and methods) and are over 2-fold significantly differentially regulated in the *ampR* mutant (Table 4). This suggests that AmpR regulates a proportion of the genes through intermediate transcriptional regulators.

**Table 4:** Transcriptional regulators and sigma factors regulated by AmpR.

Transcriptional regulators				
Locus Tag	Gene Name	Product Name	Corrected *p*-value	Fold change
PA0463	*creB*	two-component response regulator CreB	8.39E-03	−2.1
PA0479		probable transcriptional regulator	9.90E-03	−2.1
PA0610	*prtN*	transcriptional regulator PrtN	3.81E-03	11.6
PA0611	*prtR*	transcriptional regulator PrtR	6.10E-03	−3.9
PA0612	*ptrB*	repressor, PtrB	3.44E-03	19.5
PA1196		probable transcriptional regulator	9.89E-03	2.6
PA1663		probable transcriptional regulator	5.13E-03	−3.9
PA1707	*pcrH*	regulatory protein PcrH	3.98E-03	−3.8
PA2281		probable transcriptional regulator	9.89E-03	−2.1
PA2588		probable transcriptional regulator	3.77E-03	−27.9
PA3508		probable transcriptional regulator	1.60E-03	3.5
PA3604		probable two-component response regulator	6.96E-03	−2.2
PA4296	*pprB*	two-component response regulator, PprB	6.01E-03	7.4
PA4781		probable two-component response regulator	9.10E-03	2.8
PA4853	*fis*	DNA-binding protein Fis	6.65E-03	−3.3
PA5059		probable transcriptional regulator	6.33E-03	4.0
PA5105	*hutC*	histidine utilization repressor HutC	8.27E-03	−3.1
PA5380	*gbdR*	GbdR	5.75E-03	2.6
PA5483	*algB*	two-component response regulator AlgB	7.83E-03	2.4

Sigma factors				
Locus Tag	Gene Name	Product Name	Corrected *p*-value	Fold change
PA0762	*algT/U*	sigma factor AlgU	3.35E-03	5.4
PA1300		probable sigma-70 factor, ECF subfamily	9.59E-03	2.5
PA3622	*rpoS*	sigma factor RpoS	6.65E-03	2.2

AmpR influences the expression of other transcriptional regulators and sigma factors in *P. aeruginosa* PAO1, as seen in the microarray analyses. A negative sign in the fold change column indicates downregulation. ORF annotations are from the *Pseudomonas* Genome database [31].

**Figure 10:**
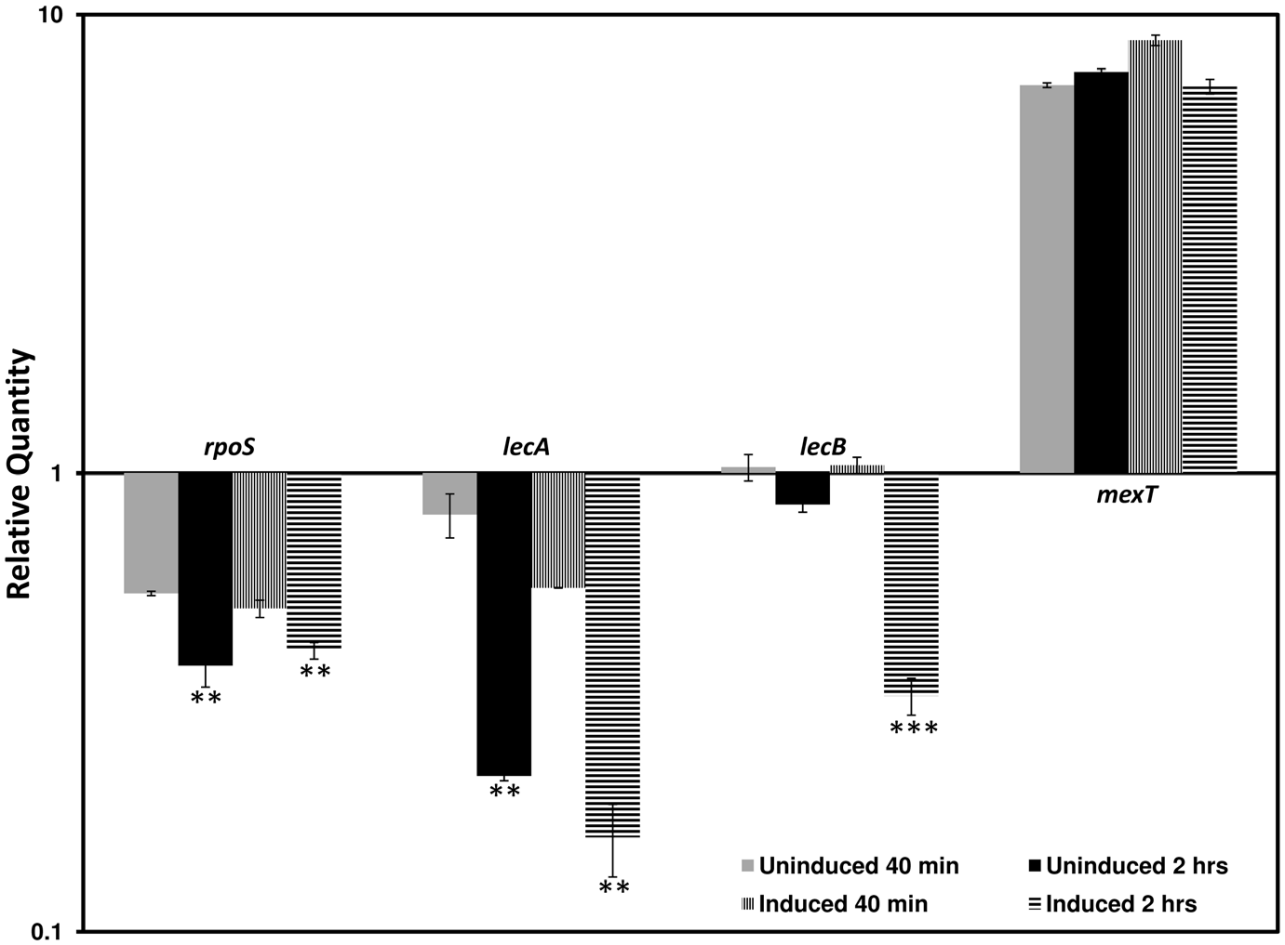
Gene expression in PAOΔ*ampR* at 40 minutes and 2 hrs post-β-lactam exposure. RNA was isolated from PAO1 and PAOΔ*ampR* cells exposed to β-lactams for either 40 minutes or 2 hours and reverse transcribed to cDNA. The expression of the sigma factor *rpoS*, *lecA* and *lecB* (galactophilic lectin genes known to be RpoS-regulated), and *mexT* (MexEF-OprN efflux pump regulator that is not RpoS-regulated) were tested by qPCR with gene-specific primers, as described in the text. Values have been normalized to expression in PAO1 under the respective conditions. ** *p*-value<0.006; *** *p*-value 0.0002 as determined by unpaired *t* test.

Three of the 24-known/putative sigma factors [88] are also over 2-fold differentially regulated, including RpoS and AlgT/U (Table 4). The stationary phase/stress sigma factor, RpoS controls virulence in different bacteria including *P. aeruginosa* [89], [90], [91]. Since *rpoS* expression is upregulated in the stationary phase [88], [90] and the RNA used for microarray analysis was harvested two hours post-β-lactam induction (at OD ∼3.0), some of the phenotypic changes seen in PAOΔ*ampR* is likely to be RpoS-mediated. To investigate this possibility, RNA was harvested 40 minutes post-induction (OD600 of ∼1.0) and the expression of known RpoS-dependent and RpoS-independent genes was compared between PAO1 and PAOΔ*ampR*. As expected, RpoS expression was significantly higher at 2 hours than at 40 minutes in PAOΔ*ampR* compared to PAO1 (*p*-value: uninduced 0.0049, induced 0.0023; Fig. 10), and this increase in RpoS expression corresponded with a growth phase-dependent increase in the expression of two of the RpoS-dependent genes, *lecA* (*p*-value: uninduced 0.0061; induced 0.0043) and *lecB* (*p*-value: uninduced NS; induced 0.0002) (Fig. 10). However, expression of the MexEF-OprN activator, MexT, which is regulated in an RpoS-independent manner, did not change at the different time points tested (Fig. 10). This suggests that AmpR, via RpoS, regulates genes of the RpoS regulon in *P. aeruginosa* in a growth phase- and stress-dependent manner, which is in agreement with previous studies [90], [91]. Moreover, the MexT data suggests that harvesting the cells either after 40 minutes or 2 hours of β-lactam treatment does not affect AmpR-mediated gene expression for non-RpoS-dependent genes (Fig. 10).

The sigma factor AlgT/U is a master regulator of alginate biosynthesis [92], [93] and we have shown previously with an *ampR* insertion mutant that there is crosstalk between AmpR and AlgT/U [40]. Our microarray data shows upregulation of AlgT/U in PAOΔ*ampR* (5.4-fold, corrected *p*-value 3.35E-03), indicating negative AmpR regulation (Table 4) in agreement with previous findings [40].

### K. Subtractive transcriptomics of the AmpR regulon

**Figure 11:**
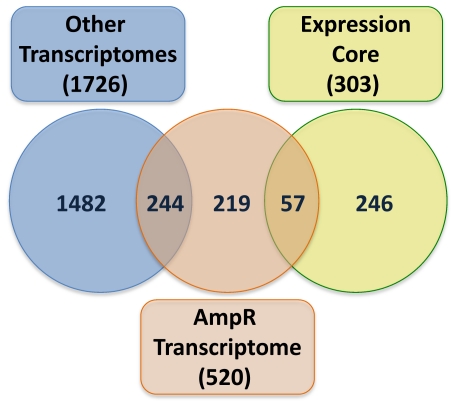
Comparison of the AmpR transcriptome with other transcriptomes. AmpR-dependent and AmpR-β-lactam-dependent genes were compared with the 303 genes of the expression core and the 1726 condition-specific genes identified previously as part of a meta-analysis of 18 *P. aeruginosa* transcriptomes [57].

Meta analysis of 18 condition-specific *P. aeruginosa* transcriptomes led to the identification of an expression core gene set of 303 genes that are significantly differentially regulated under all the different conditions analyzed and were proposed to be involved in maintaining cell homeostasis [57]. Comparing the 520 genes whose regulation is AmpR-dependent to the core led to the identification of 57 genes (of the 520) that were part of the expression core genes (Fig. 11). Further, we wanted to identify genes that are specifically under AmpR-regulation and those that are not involved in other pathways. To derive this list, we compared the 463 AmpR-dependent genes (520 minus 57 core genes) with the 1726 genes that are regulated in other conditions/by other regulators (1598 condition-specific genes [57], and 128 genes that are either RpoS [94] or AlgT/U-regulated [95]). This comparison reduced the number of AmpR-dependent genes to 133 (from 313) and the AmpR-β-lactam-dependent genes to 86 (from 207) (Fig. 11; Table S8).

The exclusively AmpR-dependent genes include the O antigen chain regulator *wzz*, the *pca* genes involved in carbon compound catabolism and *creB*, which codes for the response regulator of the CreBC TCS (Table S8). The CreBC TCS has been demonstrated to be involved in β-lactam resistance in *Aeromonas* spp. [96]. CreBC positively regulates expression of an inner membrane protein CreD in *E. coli* [97] and in *P. aeruginosa* the CreBCD system contributes to β-lactam resistance only in a ΔPBP4 background [98]. Microarray analysis of PAOΔ*ampR* in the presence β-lactam stress shows reduced *creB* expression (−2.1, corrected *p*-value 8.4E-03). Expression of *creD*, however, is significantly increased in PAOΔ*ampR* compared to PAO1 under β-lactam stress as determined by qPCR (RQ: induced 11.04±0.0001, *p*-value<0.0001). This is in agreement with a previous report that also showed *creD* overexpression in cefoxitin-treated PAOΔ*ampR* cells [98]. AmpR thus positively regulates *creB* while negatively regulating *creD* expression suggesting potential AmpR regulation of *creD* in a CreB-independent manner.

### L. Phenotypic microarray analysis of PAOΔampR

Since loss of *ampR* led to dysregulation of over 500 genes as seen in DNA microarrays, we decided to characterize its metabolic effect using phenotypic microarrays. Biolog analysis was performed with PAO1 and PAOΔ*ampR* in the absence of antibiotic stress. In all, seven phenotypes were gained and 47 phenotypes were lost (Table S9). PAOΔ*ampR* grew marginally better in media containing nutritional supplements including citrulline, histidine, shikimic acid, leucine, serine, spermidine and pyridoxal. This indicates that AmpR is a negative regulator of utilization of these agents. Of the 47 observed phenotypes that were lost in PAOΔ*ampR*, 45 belong to the sensitivity panel. Fourteen of the 45 observed phenotypes were associated with antibiotics, further supporting the role of AmpR as a major regulator of antibiotic resistance in *P. aeruginosa*.

## Discussion

*P. aeruginosa* AmpR, a LysR-type transcriptional regulator and a positive regulator of the chromosomal *ampC* β-lactamase expression, has been shown previously to play a role in regulating a few QS-dependent phenotypes and the alginate master regulator, AlgT/U [21], [40]. In this study, we determined the whole genome expression profiles of a clean in-frame deletion mutant of *ampR* in *P. aeruginosa* PAO1 under normal conditions and under sub-MIC β-lactam stress, using DNA microarrays. The results demonstrate that AmpR influences the expression of 313 genes in the absence of β-lactam stress and an additional 207 genes when exposed to sub-MIC β-lactam stress. The AmpR regulon is thus much more extensive than previously thought including virulence, antibiotic-resistance and metabolic genes.

**Figure 12:**
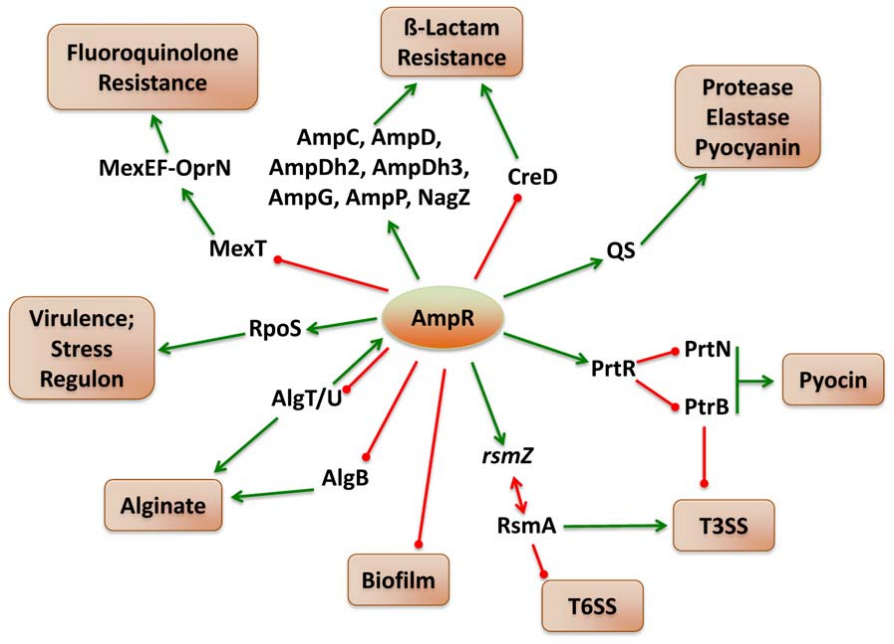
AmpR is a master regulator of gene expression in *P. aeruginosa* PAO1. AmpR positively regulates resistance to β-lactam antibiotics by upregulating expression of the *amp* genes, *nagZ* and downregulating *creD*. In addition, AmpR affects fluoroquinolone resistance by negatively regulating expression of *mexT*, the positive regulator of the MexEF-OprN efflux pump. Expression of the virulence and stress response sigma factor, RpoS and QS-regulated acute virulence factors is downregulated in PAOΔ*ampR*, indicating positive AmpR regulation. AmpR also negatively regulates biofilm formation via an unknown mechanism. AmpR modulates levels of the small RNA *rsmZ*, whose levels are lower in PAOΔ*ampR* with a corresponding enhanced expression of RsmA. Downregulation of some of the T3SS genes in the *ampR* mutant is possibly by regulating *ptrB* expression, via PrtR. Further, two major regulators of the alginate biosynthetic pathway, AlgT/U and AlgB are negatively regulated by AmpR, thereby potentially also regulating alginate production. Whether these AmpR interactions are direct or indirect needs to be investigated.

Multi-drug resistant *P. aeruginosa* isolates are a frequent occurrence in many acute and chronic infections [99]. β-lactamases and efflux pumps are major mediators of antibiotic resistance in *P. aeruginosa* [26]. We show that in addition to positively regulating the *ampC* and potentially the MexAB-OprM efflux pump by modulating expression of the MexR repressor, AmpR also mediates non-β-lactam resistance by negatively regulating the MexEF-OprN efflux pump. The PAO1 strain used for constructing PAOΔ*ampR* was the strain used in the genome-sequencing project [42], which has an 8 bp insertion in the MexEF-OprN activator *mexT* leading to premature *mexT* termination [100] and consequent non-inducibility of the MexEF efflux pump. Strains with a *nfxC* mutation, however, have different ways to overcome this, including secondary mutations and deletion of the 8 bp insertion [45], [100], leading to activation of the MexEF-OprN efflux. This was also observed in *nfxC* mutants isolated in a mouse model [101]. However, there was no differential expression of the MexT activator in both these studies [100], [101]. With PAOΔ*ampR*, even though *mexT* expression is upregulated in a β-lactam-independent manner, this will still not be able to overcome the effect of the 8 bp deletion. It has, however been suggested that there is a putative LacI-like repressor binding site in the *mexT*-*mexE* intergenic region [102] and that there is a second repressor that binds this site regulating expression of *mexEF-oprN* [103]. This suggests that the LTTR AmpR potentially regulates this LacI-type repressor, leading to induction of the MexEF-OprN pump in a MexT-independent manner. The outer membrane porin OprD serves as a conduit for the entry of carbapenems into the cell [46]. Although we see decreased expression of *oprD*, the strain is still sensitive to imipenem and meropenem, which is contrary to expectation. However, our finding is in agreement with a previous observation where *mexEF-oprN* overexpressing strains showed no altered imipenem susceptibility [104], the associated mechanism remains to be elucidated. Previous studies have also demonstrated an inverse correlation between β-lactam resistance and biofilm formation, both *in vitro* and in CF isolates [105], [106], [107]. Our data supports these findings as far as β-lactam antibiotics are concerned, since AmpR positively regulates production of AmpC β-lactamase while negatively regulating biofilm formation. However, negative regulation of the MexEF-OprN efflux (providing resistance to fluoroquinolones, chloramphenicol and trimethoprim) by AmpR (Table 2, Fig. 12) suggests that the antagonistic regulation of antibiotic resistance and biofilm formation is dependent on the class of antibiotics. The physiological advantage to the bacteria in this context is unclear. Co-regulation of β-lactam and fluoroquinolone resistance by AmpR is significant in itself, since this puts AmpR among one of the few proteins that regulates resistance to multiple classes of antibiotic [108]. In addition, since fluoroquinolones are part of the current systemic antibiotic treatment regimen for *P. aeruginosa* infections [99], this finding could potentially have important therapeutic implications.

QS is at the heart of the virulence regulatory network in *P. aeruginosa* with multiple regulators feeding into the regulation process [109]. We have shown previously that AmpR is also part of the QS regulatory process and regulates production of proteases and pyocyanin [21], [40], but the determined extent of the regulation was limited due to the experimental approaches adopted. Using whole genome transcriptome, we show here that the AmpR-influenced QS regulon is much more extensive than previously thought (Table S5). QS regulated phenotypes, such as pyocyanin and protease production are positively regulated by AmpR in the current study (Fig. 12), which is in contrast to that seen in our previous analyses [21]. We believe that this difference stems from the fact that in our previous studies, we used an insertion mutant (*aacC1* cassette inserted into the *Pst*I site of *ampR*) whereas in this study, we have used a clean in-frame deletion mutant of *ampR*. One potential reason for the discrepancy may be that the gentamycin cassette insertion at the *Pst*I site (554 bases into the *ampR* coding region) [21] did not disrupt the N-terminus HTH motif of AmpR. Since LysR members are known to bind DNA even in the absence of inducer signals [110], this intact HTH motif might have somehow interfered with the regulatory process. The *P. aeruginosa* PAO1 insertion *ampR* mutant in the previous study produced more pyocyanin compared to the wild-type PAO1 [21]. In contrast, in this study, we find that PAOΔ*ampR* produces significantly lower amounts of phenazines as compared to PAO1 (Table 3). The differences in phenazine production were also translated into differential susceptibilities in the *C. elegans* paralytic assays (Fig. 2; [40]) since phenazines are one of the major contributors to *C. elegans* toxicity in this assay [111] explaining the reduced killing of *C. elegans* with this strain (Fig. 2). Furthermore, with PAOΔ*ampR*, the microarray, qPCR and phenotypic data concur, and support our current findings.

The MexGHI-OpmD (PA4205–PA4208) efflux pump is involved in the efflux of acriflavin, ethidium bromide, novobiocin, rhodamine, and vanadium, and in maintaining QS homeostasis [60]. Mutants of *mexI* and *opmD* have been demonstrated to be impaired in QS-related phenotypes including swarming motility, production of elastase, rhamnolipids, pyocyanin and pyoverdine [60]. This pump is downregulated in PAOΔ*ampR* indicating positive AmpR regulation and correlates with some of the observed phenotypes, such as decreased production of elastase and pyocyanin. Furthermore, SoxR is known to regulate this pump [112], but is not differentially regulated in the microarray data.

AlgR regulates the *hcnABC* operon genes (*PA2193*–*PA2195*) involved in hydrogen cyanide synthesis in *P. aeruginosa* [113]. In the *ampR* mutant, these genes are downregulated five- to seven-fold without a corresponding significant differential regulation of *algR* (<two-fold). AmpR, however, negatively regulates AlgT/U (5.4-fold upregulated in PAOΔ*ampR* in the absence of antibiotics) in agreement with previous findings [40], and AlgT/U regulates *algR* [114], [115]. Thus the regulation of the *hcnABC* operon in PAOΔ*ampR* is potentially through AlgT/U-mediated regulation of AlgR (Fig. 12). Moreover, RpoS, in conjunction with the Gac-Rsm regulatory system, has been shown to regulate oxidative stress-mediated resistance in *P. fluorescens* [116]. In our study, both RpoS and the sRNA, *rsmZ* are regulated in an AmpR-β-lactam-dependent and AmpR-dependent manner, respectively. This could also be a potential mode of regulation of the *hcn* operon in PAOΔ*ampR*, provided the regulatory mechanism is conserved in *P. aeruginosa*. AmpR also negatively influences expression of the galactophilic lectin LecA (PA2570) (Fig. 10) that is RpoS and QS-regulated. LecA has been shown to be critical to the gut pathogenicity of *P. aeruginosa* and to enhance cytotoxic effects of exotoxins by inducing a permeability defect [117]. These results further support the hypothesis that AmpR regulates virulence in *P. aeruginosa*.

*P. aeruginosa* produces bacteriocins called pyocins that kill susceptible cells through either pore-formation and subsequent leakage of cytoplasmic contents, or by endonuclease activity [86]. The two high molecular weight pyocins, types R and F, are proposed to be remnants of lysogenic phages and resemble phage tails. Like lysogenic phages, they are induced in response to DNA damage, linked to the RecA-mediated SOS response [118] and other stress conditions such as exposure to hydrogen peroxide [119] or ciprofloxacin [120]. AmpR influences the expression of many of these genes including the regulators PrtR, PtrN, PtrB, and the SOS response mediator RecA (Table 4; section I in results). Specific and significant differential regulation of these genes under β-lactam stress in PAOΔ*ampR* implies a role for AmpR in influencing expression of these genes under stress conditions. Moreover, pyocin production confers a survival advantage by killing neighboring susceptible cells and 97% of the *P. aeruginosa* CF isolates tested showed bacteriocin-like killing activity [121]. The pyocin genes are located in RGP03 and RGP04 and are acquired by *P. aeruginosa* [43]. Though some RGP loci contain regulators that control expression of RGP genes [122], it is interesting to note that an endogenous regulator such as AmpR is able to regulate acquired genes and highlights the transcriptional versatility of *P. aeruginosa*.

Negative regulation of the genes involved in biofilm formation and pyocin biosynthesis by AmpR (Fig. 12) fits with the profile of AmpR as a negative regulator of chronic infection phenotypes and positive regulator of acute infection, as seen with the QS-regulated phenotypes. RsmA, a small RNA-binding protein, is a global regulator of virulence in *P. aeruginosa* and is intricately tied in with the GacSA TCS [82], [83]. Two small RNAs, *rsmZ* and *rsmY*, which bind and sequester RsmA, keep RsmA activity in check in *Pseudomonas* and other bacteria [83], [123], [124], [125], [126]. In *P. aeruginosa*, RsmA positively regulates T3SS and negatively regulates biofilm formation [127], [128]. AmpR positively regulates *rsmZ* (downregulated in PAOΔ*ampR*), resulting in downregulation of RsmA (upregulated in PAOΔ*ampR*). PAOΔ*ampR* forms enhanced biofilms (Fig. 8) and shows decreased production of QS regulated phenotypes compared to PAO1 (Table 3). These data suggest that regulation of some of the phenotypes seen in an *ampR* mutant is not via modulating the activity of *rsmZ* and consequently RsmA. However, analysis of the *rsmZ* promoter for potential AmpR binding sites using a previously identified putative AmpR binding site [51] revealed a weak AmpR consensus (5′ CCCGCGCCTTTTGT 3′). The possibility of direct AmpR regulation of *rsmZ* remains to be elucidated.

In conclusion, unlike AmpR found in other Enterobacteriaceae members, the targets of *P. aeruginosa* AmpR are widely distributed in the genome and consist of over 500 genes. Since the genes regulated include transcriptional regulators, sigma factors and non-coding RNAs, a subset of these genes are possibly regulated indirectly by AmpR via intermediate regulators. It is also interesting to note that AmpR regulates genes that have been acquired by horizontal gene transfer, which reflects on the transcriptional versatility of *P. aeruginosa*. Although the exact AmpR regulatory mechanisms are as yet unclear, this study highlights the complexity and multi-tiered regulatory processes by which *P. aeruginosa* controls expression of genes of diverse functions. Teasing apart the AmpR regulatory network will involve differentiating direct and indirect AmpR-regulated genes that will advance our understanding of how this bacterium regulates multiple different pathogenesis and metabolic mechanisms. This understanding will potentially lead to identifying mechanistic targets that can help in dealing with intractable *P. aeruginosa* infections, and other bacterial pathogens that harbor similar systems.

## Materials and Methods

### Bacterial strains, nematodes, media and primers

All experiments were performed with either the wild type or derivatives of *P. aeruginosa* PAO1 [42] and *Escherichia coli* DH5α cultivated in Luria Bertani (LB) broth (Difco, USA) incubated at 37°C unless specified otherwise. The strains and plasmids used in this study are listed in Table 5. Nematode assays were performed with *Caenorhabditis elegans* strain N2 (Bristol) and *E. coli* OP50 obtained from the Caenorhabditis Genetics Center, which is funded by the NIH National Center for Research Resources (NCRR). *E. coli* OP50 was grown in nematode growth medium (1.7% agar, 0.35% peptone, 0.34% K_2_HPO_3_, 0.3% NaCl, 0.012% MgSO_4_, 0.011% CaCl_2_, 0.0005% cholesterol) whereas the *P. aeruginosa* strains used in the nematode assays were grown on brain-heart infusion agar (Difco, USA). Biofilm assays were performed in T-broth (tryptone 10.0 g/L, NaCl 5.0 g/L). T-agar plates were prepared by addition of 1% agar to T-broth. MinS-NTA minimal media [129] was used for RNA isolation to determine expression of T3SS genes. Cation-adjusted Mueller Hinton broth and agar (Difco, USA) was used in MIC assays. For pyocyanin production, strains were cultivated in King A medium (Difco, USA). Antibiotics were supplemented, when needed, at the following concentrations for *E. coli*: ampicillin (Ap) 100 µg/ml, gentamycin (Gm) 15 µg/ml, tetracycline (Tc) 15 µg/ml; for *P. aeruginosa*: Gm 75 µg/ml, Tc 60 µg/ml. Primers used in this study are listed in Table S10.

**Table 5:** Strains and plasmids used in this study.

Strain/plasmid	Relevant characteristics	Source
**Strains**		
*Escherichia coli*		
DH5α	General purpose cloning strain; Δ*(lacZ)M15*	New England Biolabs
OP50	Food source for culturing *C. elegans*	*Caenorhabditis* Genetics Center
DBS7	DH5α harboring pEXG2 carrying the 1520 bp Δ*ampR* fragment (pDBS7)	This study
DBS220	DH5α harboring the 944 bp promoterless *ampR* ORF cloned into pCR2.1 TOPO (pDBS220)	This study
DBS271	DH5α containing the 944 bp *ampR* ORF subcloned as a *Eco*RI-*Sac*I fragment from pDBS220 into pMMB67EHGm	This study
*Pseudomonas aeruginosa*		
PAO1	Wild-type	[42]
PKM314	PAO1 with pMMB67EH-Gm (empty vector)	This study
PKM315	PAOΔ*ampR*; In-frame deletion of *ampR* (*PA4109*)	This study
PKM316	PAOΔ*ampR* with pMMB67EH-Gm (empty vector)	This study
PKM317	PAOΔ*ampR* (pAmpR); *ampR* ORF on pMMB67EH-Gm (pDBS271) moved into PAOΔ*ampR*; IPTG-inducible; Gm^R^; *ampR* complementing clone	This study
PAO*retS*::*aacC1*	Gm insertion mutant of *retS*; hyper biofilm-former	S. Lory
PAOΔ*pel*Δ*psl*Δ*algD*	In-frame triple deletion of the *algD* ORF and *pel* and *psl* operons; biofilm non-former	S. Lory
*Staphylococcus aureus*		
DBS116	Wild type *S. aureus* used for LasA assays	Lab collection
**Plasmids**		
pCR2.1 TOPO	TA cloning vector for PCR products; Ap^R^, Km^R^; ColE1 f1 *ori lacZα*	Invitrogen
pRK600	Helper plasmid for conjugation	[133]
pEXG2	Allelic exchange vector; Gm^R^, *colE1 ori*, *oriT*, *sacB*	[131]
pMMB67EH-Gm	Gm^R^; IncQ, RSF1010, lacI_q_ P*_tac_* expression vector	[134]
pDBS7	pEXG2 with the Δ*ampR* fragment, flanked by *Eco*RI and *Bam*HI sites	This study
pDBS220	pCR2.1 TOPO containing the 944 bp *ampR* ORF, PCR amplified from the PAO1 genome using DBS_*ampR*F2 and DBS_*ampR*R2	This study
pDBS271	pMMB67EH-Gm containing the *ampR* ORF, subcloned as an *Eco*RI-*Sac*I fragment from pDBS220	This study

### Construction of deletion mutants, complementation clones

An unmarked *ampR* null mutant of *P. aeruginosa* was generated by gene splicing [130] using primers KM*ampR*UF1 and KM*ampR*UR1 (to generate the upstream product P1, flanked by *Eco*RI and *Nhe*I sites), and primers KM*ampR*DF1 and KM*ampR*DR2 (to generate the downstream product P2, flanked by *Nhe*I and *Bam*HI sites). After sequencing to ensure absence of mutations, P1 and P2 were spliced together to obtain a 1520 bp deletion fragment of *ampR* containing stop codons in all three reading frames at their junction (inserted as part of the *Nhe*I sites in the primer). This was then sequenced and subcloned into a *P. aeruginosa* non-replicative plasmid pEXG2 [131] as an *Eco*RI-*Bam*HI fragment and moved into PAO1 by allelic exchange [132] using pRK600 [133] as the helper plasmid. Double crossover mutants were selected for the loss of plasmid (gentamycin-sensitive, sucrose counter-selection). The presence of deletion in PAOΔ*ampR* was confirmed using standard molecular methods (PCR and restriction analysis of amplicons) and biological assays (antibiotic sensitivity pattern and β-lactamase assays). Complementation of the *ampR* deletion was achieved by amplifying the *ampR* ORF along with the *ampR*-*ampC* intergenic region using primers DBS_*ampR*F2 and DBS_*ampR*R2 and cloning into pCR2.1 TOPO using TA cloning technique. After confirming absence of mutations by sequencing, the 944 bp *ampR* ORF was moved into pMMB67EH-Gm [134] as an *Eco*RI-*Sac*I fragment. The plasmid was then moved into PAOΔ*ampR* by electroporation [135], selecting for gentamycin-resistant colonies.

### *C. elegans* virulence assay

The *P. aeruginosa* - *C. elegans* standard paralysis assay was modified from previous protocols [66]. Overnight bacterial cultures were diluted 1∶1000 and plated onto brain heart infusion agar plates. The plates were incubated for 18–24 hours at 37°C for the formation of bacterial lawns. Meanwhile, a synchronized culture of L4 stage larvae hermaphrodite Bristol N2 *C. elegans* was washed off *E. coli* OP50-seeded nematode growth medium plates using M9 media. The nematodes were centrifuged at 1300× g for two minutes and washed twice with M9 medium to remove residual *E. coli* bacteria. Thirty to 35 nematodes were then added to each of the *P. aeruginosa* bacterial lawns. Both live and paralyzed nematodes were scored at two-hour intervals for eight hours via microscopic observation. Nematodes were considered dead when they did not respond to physical stimuli. Each strain was tested in triplicate.

### RNA isolation, generation of cDNA probes, microarray experiments and data analysis

*P. aeruginosa* strains PAO1 and PAOΔ*ampR*, with and without β-lactam antibiotic treatment were used for RNA extraction. The cells were subcultured at 37°C, 300 rpm from overnight cultures grown in LB broth to an OD600 of 0.01. When the cells reached an OD600 of 0.5–0.6, they were split into two flasks. One of the flasks was treated with sub-MIC concentration of penicillin (100 µg/ml penicillin G Potassium salt, Fisher Bioreagents) for two hours. The RNA was stabilized by addition of phenol-ethanol mixture [82] prior to extraction (RNeasy Mini Kit, Qiagen) following manufacturer protocols. Residual DNA was removed using RQ1 RNase-free DNase (Promega) and the RNA repurified using the kit. After quantification by measuring absorbance at 260 nm (Beckman DU640, Beckman Coulter), and preliminary quality check on a denaturing agarose gel (NorthernMax Gly, Ambion), the RNA integrity was analyzed using RNA Nano chips in an Agilent Bioanalyzer 2100. cDNA was then synthesized by annealing NS5 random primers to total purified RNA, spiked with *Bacillus subtilis* internal control RNAs and subsequent extension carried out using SuperScript III reverse transcriptase (Invitrogen). Fragmentation of cDNA was performed using RQ1 DNase (Promega), and the fragments were biotin labeled. Hybridization with GeneChip *P. aeruginosa* genome array (Affymetrix) and scanning were performed according to manufacturer specifications.

Data analyses from three biological replicates for each of the conditions tested were performed after normalizing and summarizing probe level measurements using Guanine Cytosine - Robust Multiarray Average (GC-RMA). Only genes that fit stringent criteria (expression cutoff: 50–100% stringency; *p*-value≤0.01 of one-way ANOVA data corrected by Benjamini Hochberg FDR; fold-change ≥2.0) were selected for further analysis. All microarray data analysis was performed using GeneSpring GX 10.0 Expression Analysis software (Silicon Genetics). The microarray data is MIAME compliant and has been deposited in NCBI GEO (accession number GSE33188).

### *Bioinformatics analysis of the AmpR-binding site*

The promoters of AmpR-dependent and AmpR-β-lactam-dependent genes (listed in Tables S3 and S4) were used to refine the AmpR binding motif using the Iterative Enhancement of Motifs (IEM) algorithm [51]. The AmpR binding site in the promoter of *ampC* (5′-TCTGCTCCAAATTT-3′) was used to search the AmpR-dependent or AmpR-β-lactam-dependent promoters from *P. aeruginosa* PAO1 and their orthologs from *P. aeruginosa* strains PA14, PA2192, C3719 and PACS2. The output of IEM was a motif matrix. WebLogo [136] was used to graphically represent the multiple sequence alignment of the output. The Regulatory Sequence Analysis Matrix Scan Tool (RSA) [137] was then used to identify PAO1 promoter sequences containing the identified AmpR-dependent or AmpR-β-lactam-dependent motifs in the respective gene sets. The RSA output was then used to generate a WebLogo for the gene sets.

### Quantitative real-time PCR

Specific genes that were significantly, over two-fold up or down regulated between *P. aeruginosa* PAO1 and PAOΔ*ampR* as seen in microarray experiments were verified by qPCR. Total RNA isolation and reverse transcription into cDNA was as described for the microarrays without addition of spike transcripts, fragmentation or labeling. For qPCR, the ABI Step One (Applied Biosystems) cycler was used with PowerSYBR Green PCR MasterMix with ROX (Applied Biosystems). Expression was normalized to *clpX* (*PA1802*), whose expression was determined to remain constant between the samples and conditions tested. Assays were performed at least in biological triplicate, each with technical triplicates, for every gene analyzed. Melt curves were determined to ensure primer specificity. The cycling conditions used were 95°C/2 minutes (holding); 40 cycles of 95°C/15 sec, 60°C/1 min (cycling); 95°C/15 sec, 60°C/1 min, 95°C/15 sec (0.6°C ramp) (melt curve). Differential regulation of the T3SS genes, including *rsmZ* was determined using RNA isolated from cells grown in MinS-NTA inducing media [129] from PAO1 and PAOΔ*ampR*, essentially as described above.

Gene expression in PAOΔ*ampR* were normalized to the corresponding PAO1 values, for both the β-lactam uninduced and induced conditions and is presented as relative expression in PAOΔ*ampR* ± standard error.

### Determination of minimum inhibitory concentration (MIC)

MICs were determined one of two ways. For testing the MIC of β-lactams, E-test was used following manufacturer protocols (BioMerieux). The MICs of the MexEF-OprN efflux pump substrates (ofloxacin, chloramphenicol, ciprofloxacin, and trimethoprim) were determined by standard broth microdilution method [50]. Briefly, serial two-fold dilutions of the antibiotics were incubated at 37°C for 16–18 hrs with 5×10^5^ CFU/ml of bacteria in a total volume of 200 µl in 96-well flat bottom polystyrene plates. The highest dilution of antibiotic that prevented bacterial growth was considered as the MIC. The assays were performed at least in triplicate, each with technical triplicate, for each antibiotic in cation-adjusted Mueller Hinton broth.

### Quantifying β-lactamase activity

β-lactamase activity was quantified as described previously [21]. Briefly, cells in LB broth at an OD600 of 0.5–0.6 were treated with 100 µg/ml Penicillin G for two hours at 37°C. The cells were then harvested, OD normalized, and lysed with BugBuster Protein Extraction Reagent (Novagen) and r-Lysozyme (Novagen), and treated with Benzonase nuclease (Novagen). The amount of β-lactamase was quantified in the soluble fraction by determining hydrolyzing activity on nitrocefin (Oxoid, England). Protein concentrations in the samples were determined by Bradford assay. Enzyme activity was expressed as milliunits of β-lactamase (nanomoles of nitrocefin hydrolyzed per minute per microgram of protein).

### Biofilm assays

Time course biofilm assays were performed in 12×75 mm round-bottom glass culture tubes (VWR) as described previously [75]. Briefly, fresh overnight T-agar plate cultures of the test strains were scraped into 1 ml T-broth and diluted to a final OD600 of 0.0025. Aliquots of 1 ml per tube were made at time zero and incubated static at room temperature for 24, 48, 72 and 96 hours. To assay for pellicle formation, the tubes were washed with running tap water five times, after discarding the cultures and stained with 1% crystal violet for 20 minutes. After pouring off the dye, the tubes were washed thoroughly with running tap water 10 to 15 times. Quantification of the attached and stained cells was done at 590 nm after solubilization of the dye with absolute ethanol.

### Motility assays

Twitching and swimming assays were performed on 1% and 0.3% agar plates, respectively, as described previously [138].

### Protease assays

LasA protease activity was measured by the ability of the strain supernatant to lyse boiled *Staphylococcus aureus* cells as described [139]. Overnight culture supernatants of the test strains (100 µl) were mixed with 900 µl of a Tris-HCl (pH 8.5) suspension of boiled *S. aureus* culture diluted to a final OD600 of 0.8. The lysis was monitored over an hour and LasA activity was expressed as the change in OD600 per hour per µg protein.

LasB elastolytic assay was performed with an elastin-congo red (ECR, Sigma) conjugate [140]. The overnight culture supernatants (100 µl) were mixed with 900 µl of ECR buffer (100 mM Tris, 1 mM CaCl_2_, pH 7.5) containing 20 µg of ECR. Tubes were incubated shaking for one hour at 37°C, and the supernatant was read at 495 nm. LasB activity was expressed as change in A495 per µg protein compared to an LB control.

### Pyocyanin production

The amount of pyocyanin produced was determined by extracting the pigment from overnight King A culture supernatants. A 5 ml 18-hour supernatant was mixed with 3 ml of chloroform to extract pyocyanin into the chloroform phase. Pyocyanin was then extracted with 0.2 N HCl, the absorbance measured at 520 nm, and the pyocyanin concentration expressed as µg pyocyanin produced per µg of protein [141].

### Phenotypic Microarray (PM)

PM profiles of PAOΔ*ampR* were compared to that of wild-type PAO1 in the absence of antibiotic stress to test the effect of *ampR* deletion. PM arrays (Biolog Inc., Hayward, CA, USA) comprise of about 2000 tests spanning 20 96-well plates and include ∼800 tests for carbon, nitrogen, phosphorous and sulfur utilization, ∼100 tests each for pH growth range and osmotic sensitivity, and ∼1000 tests for chemical sensitivity. Suspensions of control and test strains, in duplicate, were prepared in inoculating fluid containing 0.01% tetrazolium violet and transferred to the PM plates. After incubation for 24 hours, growth differences between the strains were determined from the kinetic response curves obtained by measuring changes in the color of the redox dye in each well in the OmniLog® incubator reader. Better growth of PAOΔ*ampR* in the presence of a specific test compound compared to PAO1 indicates gain of the phenotype (AmpR negatively regulates the phenotype) whereas poorer growth of the mutant compared to the wild type in a specific well indicates loss of phenotype due to *ampR* deletion (AmpR positively regulates the phenotype). OmniLog® PM software was used for data analysis.

### Statistical analysis

All data were analyzed for statistical significance using *t*-test on GraphPad statistical analysis software, except for the microarray data, which was performed on GeneSpring GX 10.0 as mentioned earlier.

## Supporting Information

Table S1 **Key to identifying the AmpR-dependent, and AmpR- and ß-lactam dependent genes from the venn diagram (** **Fig. 4** **).** Condition A: PAO1 uninduced vs PAO1 induced; Condition B: PAOΔ*ampR* uninduced vs PAOΔ*ampR* induced; Condition C: PAO1 uninduced vs PAOΔ*ampR* uninduced; Condition D: PAO1 induced vs PAOΔ*ampR* induced. NA- not applicable.
(XLSX)

Table S2 **ß-lactam-dependent genes.** The 206 ß-lactam stress-dependent genes are separated into upregulated and downregulated genes with the corresponding corrected *p*-values for the fold change (FC) observed. The genes are arranged based on either (A) functional categorization, or (B) fold change. Locus tag annotations are from the *Pseudomonas* Genome database [31].
(XLSX)

Table S3 **AmpR-dependent genes.** The 313 AmpR-dependent genes are separated into upregulated and downregulated genes with the corresponding corrected *p*-values for the fold change (FC) observed. The genes are arranged based on either (A) functional categorization, or (B) fold change. Locus tag annotations are from the *Pseudomonas* Genome database [31].
(XLSX)

Table S4 **AmpR, ß-lactam-dependent genes.** The 207 genes that are specifically dependent on AmpR, and ß-lactam stress are separated into upregulated and downregulated genes with the corresponding corrected *p*-values for the fold change (FC) observed. The genes are arranged based on either (A) functional categorization, or (B) fold change. Locus tag annotations are from the *Pseudomonas* Genome database [31].
(XLSX)

Table S5 **Regulation of QS-related genes by AmpR.** List of genes that are significantly differentially regulated in PAO1 and PAOΔampR in the presence and absence of ß-lactam stress. For sake of clarity, only significant fold changes (FC) and their corresponding corrected p-values in the various conditions are shown. Annotations for the locus tags are from the Pseudomonas Genome database [31].
(XLSX)

Table S6 **Regulation of biofilm-specific genes by AmpR.** Significantly differentially regulated genes in PAO1 and PAOΔampR in the presence and absence of ß-lactam antibiotics. For sake of clarity, only significant fold changes (FC) and their corresponding corrected p-values are shown. Locus tag annotations are from the Pseudomonas Genome database [31].
(XLSX)

Table S7 **Genes in RGPs that are AmpR-regulated.** AmpR regulates genes that are part of the different RGPs both in the presence and absence of a ß-lactam antibiotic, listed here with the corresponding fold changes (FC) and p-value. Locus tag annotations are from the Pseudomonas Genome database [31].
(XLSX)

Table S8 **Exclusively AmpR-, and AmpR-ß-lactam-dependent genes.** The list of 219 genes, derived from the lists in Tables S3A and 4A, that are specifically dependent on AmpR, both without and with ß-lactam stress, and are not differentially regulated in any of the 20 other published transcriptome studies (see text for details). Genes are listed as either up- or down-regulated with the corresponding corrected p-values for the fold change (FC) observed. Locus tag annotations are from the Pseudomonas Genome database [31].
(XLSX)

Table S9 **Phenotypic microarray analysis of PAO1 and PAOΔampR.** Phenotypic microarray analysis was performed using Biolog plates, as explained in the text. Phenotypes gained indicate negative regulation by AmpR of these phenotypes, whereas phenotypes lost are those that are positively regulated by AmpR.
(XLSX)

Table S10 **Primers used in this work.** All primers were designed as part of this study. qRT in the primer name indicates that the primer was designed for qPCR.
(XLSX)
